# Multicenter evaluation of low inoculum and a colorimetric assay for antifungal susceptibility testing of *Aspergillus fumigatus* to echinocandins with the EUCAST E.Def 9.4

**DOI:** 10.1128/jcm.01763-25

**Published:** 2026-05-22

**Authors:** Joseph Meletiadis, Maria Siopi, Karin Meinike Jørgensen, Pilar Escribano, Jesus Guinea, Maiken Cavling Arendrup

**Affiliations:** 1 Clinical Microbiology Laboratory, "Attikon" University General Hospital, Medical School, National and Kapodistrian University of Athens 68993, Athens, Greece; 2 Unit of Mycology, Statens Serum Institut 4326, Copenhagen, Denmark; 3 Clinical Microbiology and Infectious Diseases Department, Hospital General Universitario Gregorio Marañón, Madrid, Spain; 4 Instituto de Investigación Sanitaria Gregorio Marañón, Madrid, Spain; 5 CIBER Enfermedades Respiratorias-CIBERES (CB06/06/0058), Madrid, Spain; 6 Faculty of Health Sciences - HM Hospitals, Universidad Camilo José Cela, Madrid, Spain; 7 Department of Clinical Microbiology, University Hospital Rigshospitalet, Copenhagen, Denmark; 8 Department of Clinical Medicine, University of Copenhagen 4321, Copenhagen, Denmark; University of Utah, Salt Lake City, USA

**Keywords:** MEC, colorimetric, wild type, echinocandins, *Aspergillus*

## Abstract

Echinocandin minimal effective concentration (MEC) determination for *Aspergillus* is challenging with the EUCAST method. We investigated the applicability of a lower inoculum (10^3^ CFU/mL) and a colorimetric assay for micafungin and anidulafungin against *Aspergillus fumigatus* with the EUCAST E.Def 9.4 protocol. Eighteen shared *A. fumigatus* isolates (12 wild-type [WT] and 6 non-WT isolates) together with five local *A. fumigatus* isolates were tested (blinded to the phenotype) in three laboratories for susceptibility to anidulafungin and micafungin following the EUCAST E.Def 9.4, except using a final inoculum of 10^3^ CFU/mL. Endpoints were determined after 48 h of incubation macroscopically (MEC-0; no haziness, MEC-1; slight haziness) and colorimetrically after up to an additional 3 h of incubation with XTT/menadione (400 mg/L/6.25 μM) (MIC-1, MIC-2, and MIC-3 corresponding to 25%, 50%, and 75% color development compared to drug-free control). The inter-center reproducibility and agreement between macroscopic and colorimetric endpoints within one twofold dilution were calculated. MEC and MIC distributions were analyzed, and the WT upper limit (WT-UL) was determined. MEC-0 was reproducible (83%–100%) and best correlated with MIC-2 (67%–100%), provided that good metabolic activity was observed in the growth control (>0.5 absorbance at 450/630 nm). The WT-UL was 0.03 mg/L for macroscopic MEC-0 and colorimetric MIC-2 endpoints for both anidulafungin and micafungin. Visual MEC-0s and XTT MIC-2s of non-WT isolates were >0.125 mg/L. The low inoculum facilitated echinocandin MEC determination, and the XTT assay increased objectivity and enabled automation for the separation of WT and non-WT *A. fumigatus* isolates with the EUCAST method.

**IMPORTANCE:**

Susceptibility testing of *Aspergillus* to echinocandins is challenging with the EUCAST method because of the difficulties associated with the visual determination of the minimal effective concentration (MEC) and the lack of susceptibility interpretative criteria. We therefore conducted a multicenter study in order to define the MEC distribution of micafungin and anidulafungin against *Aspergillus fumigatus* with the EUCAST E.Def 9.4 protocol using a low inoculum of 10^3^ CFU/mL and a colorimetric assay. The MEC was reproducible (83%–100%) and best correlated with colorimetric MIC-2, corresponding to a 50% reduction of color development (67%–100%). The upper MEC and MIC-2 limits of wild-type (WT) isolates were 0.03 mg/L for both anidulafungin and micafungin, whereas non-WT isolates had MEC and MIC-2 >0.125 mg/L. The low inoculum facilitated echinocandin MEC determination, and the XTT assay increased objectivity and enabled automation for the separation of WT and non-WT *A. fumigatus* isolates with the EUCAST method.

## INTRODUCTION

Antifungal susceptibility testing of echinocandins against *Aspergillus* spp. with the EUCAST broth microdilution method E.Def 9.4 relies on visual determination of a minimum effective concentration (MEC) endpoint (1). The MEC is defined as the lowest concentration at which abnormal, short, and branched hyphal clusters (rosettes) are observed in contrast to the long, unbranched hyphal elements that are seen in the growth control well. A multicenter evaluation of E.Def 9.4 for susceptibility testing of *Aspergillus fumigatus* to echinocandins has shown that the standard inoculum (10^5^ CFU/mL) used in the EUCAST method results in dense growth, which complicates micro- and macroscopic determination of the MEC (2). Moreover, spectrophotometric readings were not reliable because of uneven growth on the bottom of the wells (2). Single-center data have indicated that modifications of the E.Def 9.4 method, by use of a lower inoculum (10^3^ CFU/mL), facilitate visual MEC determination and result in narrow MEC wild-type (WT) distributions (3). Moreover, a colorimetric assay based on XTT conversion by viable fungi in the presence of the electron coupling agent menadione (MEN) could increase objectivity and enable automation (2). We therefore conducted a multicenter study to describe the MEC distributions of micafungin and anidulafungin against *A. fumigatus* WT isolates, established upper MEC limits of the WT population (WT-UL) following the modified EUCAST broth microdilution E.Def 9.4, and assessed whether correct classification of WT and non-WT isolates could be achieved by including previously characterized echinocandin-resistant *A. fumigatus* isolates.

## RESULTS

### Macroscopic determination

**Fig 1:**
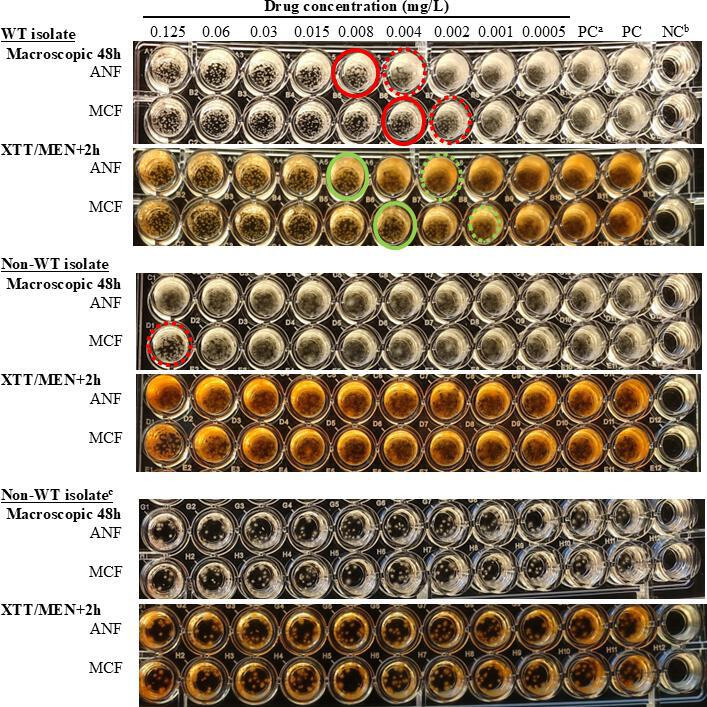
Macroscopic and colorimetric determination of echinocandin activity with EUCAST E.Def 9.4 and a lower-than-the-standard inoculum (10^3^ CFU/mL). Photos were taken using a magnifying mirror under a black background. Red solid circle: MEC-0, pinpoint mycelia in clear medium. Red dotted circle: MEC-1, pinpoint mycelia and slightly hazy medium. Green solid circle: MIC-2 after 2 h incubation with XTT/MEN. Green dotted circle: MIC-3 after 2 h incubation with XTT/MEN. ^a^PC, positive control; ^b^NC, negative control; ^c^the DPL3248 non-WT isolate grows in drug-free control as in drug-containing wells, forming pinpoint colonies.

All WT isolates presented a uniform growth pattern at sub-MEC-1 concentrations similar to that observed in the drug-free control. In contrast, at supra-MEC-0 concentrations, microcolonies in clear medium were found even at the highest concentration tested (Fig. 1). At drug concentrations between MEC-0 and MEC-1, a mixed pattern of microcolonies and uniform hazy growth was observed. For three non-WT isolates, the growth pattern was similar to that of the drug-free control (uniform growth in the entire well) even at high drug concentrations, whereas for the remaining three non-WT strains (DPL32458, DPL55985, and DPL24053), comparable microcolonies were observed also in the drug-free control, as shown in the last two photos of Fig. 1. As these isolates either harbor fks mutation or the fks enzyme is insensitive to echinocandin, and all of them were isolated from patients on echinocandin therapy, they should be considered non-WT (4, 5). This emphasizes the importance of comparison with growth in drug-free control before categorizing the phenotype of the isolate.

**Fig 2:**
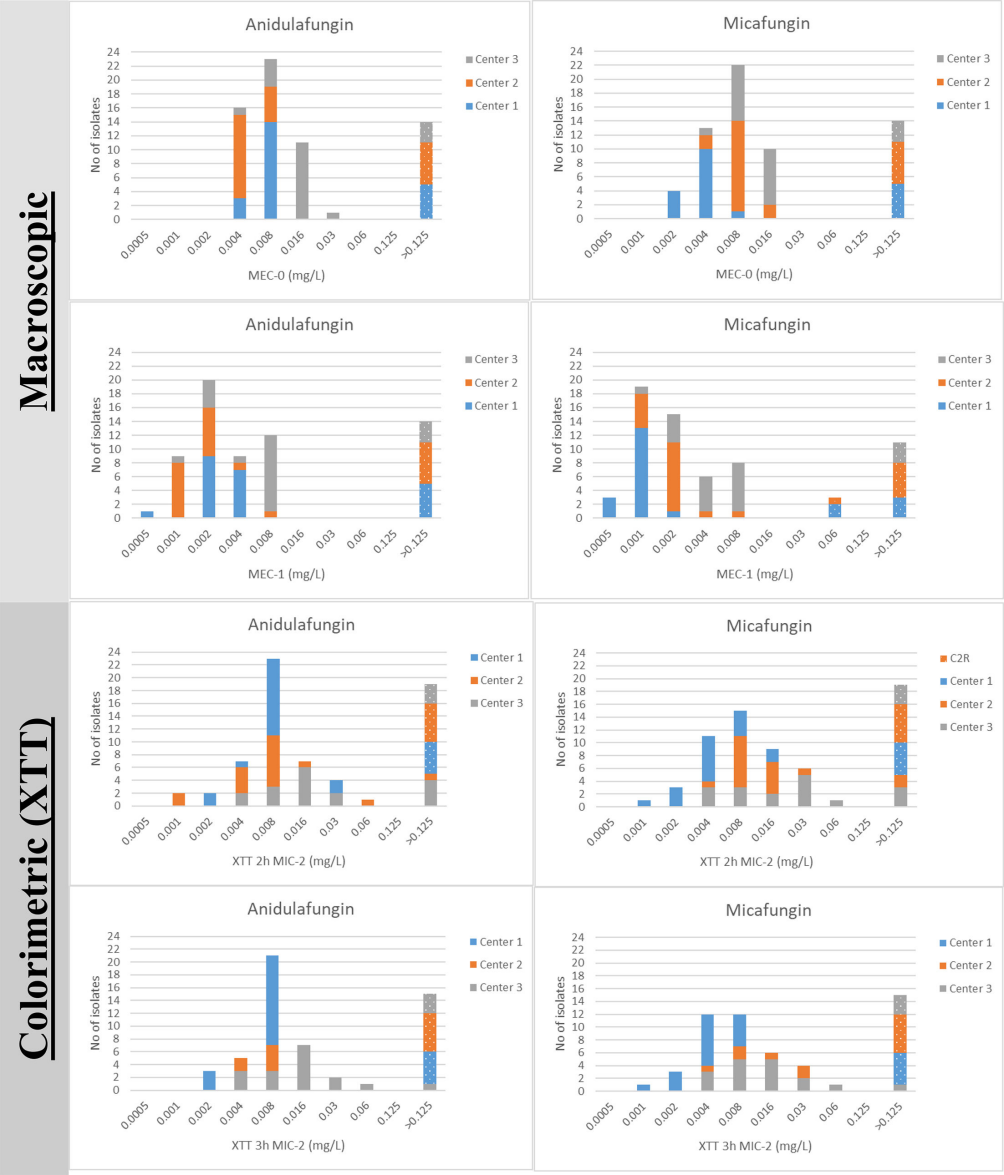
Distributions of macroscopic (MEC-0 and MEC-1) and colorimetric (MIC-2 after 2 and 3 h of incubation) endpoints for all three centers for shared and local *A. fumigatus* isolates. Shaded bars indicate non-WT isolates. No MEC and MICs could be determined for six shared and five local WT isolates in center 2 after 3 h because the XTT absorbance of growth control reached the upper limit of spectrophotometric reading, and for one non-WT isolate in center 1 and three non-WT isolates in center 3 because of contamination problems.

The median (range) MEC-0 and MEC-1 values against WT isolates are shown in Table 1. The MEC-0 and MEC-1 of WT isolates were ≤0.03 mg/L as opposed to non-WT isolates, where the MEC-0 and MEC-1 values were ≥0. 06 mg/L. The actual MEC-0s against non-WT isolates were 0.25–8 mg/L for micafungin and 1–4 mg/L for anidulafungin. Overall, the use of MEC-0 resulted in more reproducible results than MEC-1 among the centers. The median difference for all strains between MEC of each center and the median MEC among the three centers ranged from −1 to +1, with 83%–100% being within one twofold dilution for all three centers for anidulafungin and two centers for micafungin (micafungin MEC-0s in center 1 were slightly lower, with only 53% being within one twofold dilution). MEC distributions are shown in Fig. 2. The modal MEC-0 values determined at the three centers against shared and local isolates fell within one twofold dilution of the global modal MEC (Fig. 2). A WT-UL MEC-0 was determined at 0.03 mg/L for both echinocandins.

**TABLE 1:** The median (range) macroscopic MEC endpoints against the 12 WT shared isolates and differences among the three centers^*b*^

Drug	Center	Median (range) MEC-0 (mg/L)	Differences from median MEC among centers		Median (range) MEC-1 (mg/L)	Differences from median MEC among centers	
			Median (range)	% Within ±1 twofold dilution		Median (range)	% Within ±1 twofold dilution

Anidulafungin	1	0.008 (0.004–0.008)	0 (0–1)	100	0.002 (0.0005–0.004)	0 (0–2)	92
	2	0.004 (0.004–0.008)	1 (0–1)	100	0.001 (0.001–0.008)	1 (0–2)	83
	3	0.016 (0.008–0.016)	−1 (−2 to 0)	83	0.008 (0.004–0.008)	−1.5 (−3 to 0)	50
Micafungin	1	0.004 (0.001–0.008)	1 (0–3)	58	0.001 (0.0005–0.002)	1 (0–2)	75
	2	0.008 (0.004–0.016)	0 (0–0)	100	0.002 (0.001–0.008)	0 (−1 to 1)	100
	3	0.016 (0.008–0.016)	−0.5 (−2 to 0)^*a*^	92	0.008 (0.004–0.008)	−2 (−3 to 0)	42

^*a*^ Non-integer numbers can be obtained when the median is between two twofold dilutions.

^*b*^ MICs could not be determined for one isolate in center 1 and three isolates in center 3 because of contamination problems.

### Colorimetric determination

For WT isolates, color intensity decreased at higher drug concentrations, whereas for non-WT isolates, color intensity was the same across different concentrations even for the isolates with the abnormal growth pattern in the drug-free control (DPL32458, DPL55985, and DPL24053) (Fig. 1). XTT conversion after 1 h of incubation was minimal for the growth control with absorbance (ABS) < 0.5 in all centers prohibiting MIC determination. XTT conversion after 2 and 3 h of incubation was good (ABS > 0.5) and excellent (ABS > 1), respectively, for most strains. In one center, the ABS after 3 h in the growth control wells surpassed the upper detection limit of the reader (ABS > 2.5) for 6/12 shared WT isolates, thus preventing the determination of XTT 3 h MIC endpoints.

XTT MIC-2s (corresponding to 50% color inhibition) against non-WT isolates were >0.125 mg/L. For WT isolates, the XTT MIC endpoints after both 2 and 3 h of incubation resulted in reproducible results (91%–93%) among the three centers (Table 2). After 2 h, most WT isolates (28/36, 78%) had an XTT MIC-2 of ≤0.03 mg/L (Fig. 2) except 8/36 (22%) isolates, which were misclassified as non-WT, including four isolates in center 2 (three isolates with low metabolic activity, growth control ABS 0.42–0.48, and XTT MIC-2 >0.125 mg/L and one isolate with good metabolic activity, growth control ABS 1.0, and XTT MIC-2 0.06 mg/L) and four isolates in center 3 (all with good metabolic activity, growth control ABS of 0.64–0.88, and XTT MIC-2 >0.125 mg/L). For the five isolates with good metabolic activity (growth control ABS > 0.64), the XTT MIC-3s were ≤0.03 mg/L, correctly classifying them as WT. For the three isolates in center 2 with low metabolic activity at 2 h (growth control ABS 0.42–0.48), after 3 h of incubation, the metabolic activity increased to ABS 0.75–0.88 with XTT MIC-2s ≤0.03 mg/L, correctly classifying them as WT. A MIC-2 WT-UL of 0.03 mg/L was determined for both anidulafungin and micafungin.

**TABLE 2:** Inter-center reproducibility of different XTT MIC endpoints as % of MICs within one twofold dilution from the median MIC among the three centers for the 12 WT *A. fumigatus* isolates

Drug	XTT/MEN incubation (h)	MIC-1^*a*^ (%)	MIC-2^*a*^ (%)	MIC-3^*a*^ (%)
Anidulafungin	2	92	86	89
	3^*b*^	90	97	97
Micafungin	2	81	89	81
	3^*b*^	83	97	93

^*a*^ The XTT MICs were determined as the lowest drug concentration corresponding to 25% (MIC-1), 50% (MIC-2), or 75% (MIC-3) of metabolic activity compared to the growth control.

^*b*^ In one center, 6/12 reached ABS > 2.5 after 3 h, which was higher than the upper detection limit of the reader, thus not enabling determination of XTT/MEN 3 h MIC endpoints.

### Macroscopic vs colorimetric endpoints

Comparison of MEC-0 with the XTT MIC endpoints showed the best agreement with MIC-2 (67%–100%) after both 2 and 3 h (Table 3), with 3 h giving consistently higher levels of agreement than 2 h for anidulafungin (83%–100% vs 67%–75%), though at the expense that not all isolates could be read at 3 h (due to control ABS above limit). Comparison of MEC-1 with the XTT MIC endpoints showed the best agreement with MIC-3 (data not shown).

**TABLE 3:** Essential agreement (within one twofold dilution) between macroscopic MEC-0 and different XTT MIC endpoints after 2 and 3 h of incubation with XTT/MEN for each center for the 12 WT *A. fumigatus* isolates

Drug	Center	XTT/MEN incubation (h)	MIC-1^*a*^ (%)	MIC-2^*a*^ (%)	MIC-3^*a*^ (%)
Anidulafungin	1	2	25	75	50
		3	17	**92^*c*^**	33
	2	2	0	67	67
		3^*b*^	0	**100**	67
	3	2	8	67	42
		3	17	**83**	33
Micafungin	1	2	17	**83**	50
		3	8	**83**	42
	2	2	17	**67**	58
		3^*b*^	0	**67**	67
	3	2	0	**75**	42
		3	8	**75**	42

^*a*^ The XTT MICs were determined as the lowest drug concentration corresponding to 25% (MIC-1), 50% (MIC-2), or 75% (MIC-3) of metabolic activity compared to the growth control.

^*b*^ 6/12 reached ABS > 2.5 after 3 h, which was higher than the upper detection limit of the reader, thus not enabling determination of XTT/MEN 3 h MIC endpoints.

^*c*^ The largest agreement for each center are indicated in bold.

## DISCUSSION

The MEC determination against WT isolates resulted in reproducible unimodal and narrow normal distributions among the three centers in this multicenter study using the modified low (10^3^ CFU/mL) inoculum EUCAST E.Def. 9.4 protocol for echinocandin susceptibility testing of *A. fumigatus*. A WT-UL of 0.03 mg/L was determined. Based on this WT-UL, all non-WT isolates were correctly classified by all three centers. The XTT colorimetric assay can be used for MEC determination based on 50% color development compared to drug-free control after 2 h of incubation (XTT MIC-2 endpoint). XTT MIC-2 against non-WT isolates were >0.125 mg/L, whereas XTT MIC-2s against WT isolates were lower, ≤0.03 mg/L. However, their distributions for WT isolates were wider than for the visual MEC reading, and in two centers, 1 and 3 isolates, respectively, were misclassified as non-WT, adopting an XTT MIC-2 WT-UL of 0.03 mg/L. For isolates with good metabolic activity at 2 h (growth control ABS > 0.5), XTT MIC-3 should be used for classification, whereas for isolates with low metabolic activity at 2 h (growth control ABS < 0.5), a 3 h incubation time will be required to verify a WT phenotype. A spectrophotometer with a dynamic range of readings >2.5 ABS is recommended to avoid off-scale measurements.

These findings are in line with a previous single-center study where the conditions of echinocandin susceptibility testing of *Aspergillus* spp. with EUCAST E. Def 9.4 were optimized in order to facilitate MEC determination (3). This multicenter study confirmed the feasibility and reproducibility of MEC determination with EUCAST E.Def 9.4 using a low inoculum of 10^3^ CFU/mL. This modification can be easily adapted to EUCAST E.Def 9.4 with an extra dilution of 1/100 for the inoculum used for azole susceptibility testing. In light of the recent revision of EUCAST E.Def 9.4 allowing spectrophotometric readings for MIC determination of azoles and *A. fumigatus,* spectrophotometric MIC determination of echinocandins is possible after the standard 48 h incubation protocol for MIC determination with an extra 2–3 h incubation with XTT/MEN, which will allow differentiation of WT from non-WT isolates based on color development. However, in this study, visual MEC determination was simpler and superior. In addition, as XTT conversion is affected by the metabolic status of an isolate, which is affected by environmental conditions like oxygenation and humidity, and fungal biomass, significant inter-center and inter-experimental variation can be observed.

Given the indication of echinocandins as empiric therapy for presumed fungal infections and as part of combination therapy against azole-resistant aspergillosis, monitoring the susceptibility of *Aspergillus* spp. to echinocandins is important. Although few echinocandin-resistant *A. fumigatus* isolates have been described so far, they all emerged after therapy with echinocandin. The modifications of EUCAST E.Def 9.4 proposed in the present study, with the use of a low inoculum with/without XTT/MEN for echinocandin susceptibility testing of *A. fumigatus,* will hopefully promote further studies with other *Aspergillus* species and future determination of associated ECOFFs. This would allow detection of non-WT isolates, characterization of new resistance mechanisms, and monitoring of epidemiological changes.

## MATERIALS AND METHODS

### Isolates

A total of 23 molecularly identified *A. fumigatus* clinical isolates, including 18 shared isolates (showing low [*n* = 12] and high [*n* = 6] MECs and stated as WT and non-WT, respectively, for simplicity despite the absence of ECOFFs), together with five isolates from the local collection of each center, were tested in three laboratories. The five local isolates were included in order to introduce more biological variation, take into account local epidemiology in each center, and verify MEC distributions generated with the shared isolates. The six non-WT isolates kindly provided by D. Perlin (DPL) and Cornelia Lass-Flörl (AF), showing elevated MEC values, with (DPL1035-homo [4], DPL24053) or without (AF1, AF13, DPL55985, DPL32458 [6]) known *FKS* alterations (S679P for DPL10135 and F675S for DPL24053), were included. Although only a few echinocandin-resistant isolates were tested, echinocandin resistance in *A. fumigatus* is very rare and scarcely reported worldwide. *Candida krusei* ATCC 6258, *Candida parapsilosis* ATCC 22019, and *Candida albicans* CNM-CL-F8555 were used as quality control strains. The isolates were stored in normal sterile saline/oxebroth with 10% glycerol at −70°C/−80°C until use.

### Antifungal drugs, chemical reagents, and medium

Laboratory-grade standard powders of anidulafungin (Pfizer, Inc., Groton, CT) and micafungin (Astellas Pharma, Inc., Tokyo, Japan) were dissolved in sterile DMSO (Chem-Lab NV, Zedelgem, Belgium, or Merck, Søborg, Denmark), and stock solutions were prepared at 10 mg/mL and stored at −70°C or at 5 mg/mL and stored at −80°C. The 2,3-bis-(2-methoxy-4-nitro-5-sulfophenyl)-2H-tetrazolium-5-carboxanilide (XTT) sodium salt (Applichem, Darmstadt, Germany) was dissolved in sterile water before use. MEN (Sigma-Aldrich, Steinheim, Germany) was dissolved in absolute ethanol (VWR Chemicals, Fontenay-sous-Bois, France, or SSI Diagnostica, Hillerød, Denmark), and stock solutions of 58 × 10^−3^ M were stored at −70°C or −80°C. The medium used throughout was RPMI 1640 medium (with L-glutamine, without bicarbonate) (AppliChem, Darmstadt, Germany), buffered to pH 7.0 with 0.165 M MOPS (AppliChem, Darmstadt, Germany), and supplemented to a final concentration of 2% glucose (AppliChem, Darmstadt, Germany) or purchased ready-made (SSI Diagnostica, Hillerød, Denmark).

### Broth microdilution susceptibility testing

The modified reference broth microdilution procedure was carried out according to the EUCAST E.Def 9.4 protocol, but by using a lower inoculum (1). Briefly, twofold serial drug concentrations ranging from 0.0005 to 0.125 mg/L of the two echinocandins were prepared. Each isolate was revived by sub-culturing twice on Sabouraud dextrose agar plates with gentamicin and chloramphenicol (SGC2; bioMerieux) at 30°C for 5–7 days or on YGC agar plates (Fisher Scientific, Roskilde, Denmark) at 37°C for 2–3 days, and conidial suspensions were prepared in sterile water with 0.1% Tween 20 (Merck, Søborg, Denmark). Conidia were then counted in a hemocytometer or adjusted to McFarland 0.5 using an OD-meter and diluted in sterile water in order to obtain 2× the final inoculum of 10^3^ CFU/mL. Plates (tissue-treated trays, CELLSTAR reference 655 180; Greiner Bio-One, Frickenhausen, Germany, in center 1, and Thermo Scientific Nunc MicroWell 96-Well, Nunclon Delta-Treated, Flat-Bottom Microplate, Fisher Scientific Biotech Line ApS, Roskilde, in centers 2 and 3) were inoculated and incubated at 34°C to 37°C for 48 h. The inoculum sizes were confirmed using quantitative colony counts for a selected strain set. The MECs were those concentrations leading to the observation of abnormal, short, and branched hyphal clusters (rosettes) in contrast to the long, unbranched hyphal elements that are seen in the growth control well, which macroscopically appears as pinpoint mycelial colonies (no haziness in the well) (MEC-0) (Fig. 1). An additional endpoint was determined corresponding to the lowest concentration with the presence of pinpoint mycelial colonies together with healthy hyphae (slight haziness in the well) (MEC-1) (Fig. 1).

### Colorimetric method

A recently described XTT method was evaluated (2). The ABS at 450/630 nm was measured after 1, 2, and 3 h of incubation after adding 50 μL of 5× XTT/MEN solution (final concentrations 400 mg/L/6.25 μM) in each well of the broth microdilution plates used for MEC determination. The spectrophotometer Tecan F200 (JM Kyriakidis, Athens, Greece), Multiskan FC with incubator 357-713061T (Thermo Fisher, Madrid, Spain), and Biotek EPOCH2 (Halby, Brøndby, Denmark) were used in each of the three centers, respectively. The percent metabolic activity assessed by percent XTT conversion was calculated for each well as (ABS_drug well_ − ABS_background drug well_)/(ABS_GC well_ − ABS_background GC well_) × 100%, where GC is growth control. The colorimetric MICs were determined as the lowest drug concentration corresponding to 25% (MIC-1), 50% (MIC-2), or 75% (MIC-3) of metabolic activity compared to the growth control (Fig. 1).

### Analysis

The median (range) MEC and MIC values were calculated, and MEC and MIC distributions were constructed for each center. The inter-center agreement for both types of endpoint was calculated as the proportion of isolates for which MEC and MIC ranges fell within one twofold dilution from the median MEC and MIC, respectively, among the three centers. The essential agreement (within one twofold dilution) between MECs and MICs was determined for each center. The WT-UL was determined with the ECOFFinder program and the eyeball method.
